# SARS-CoV-2-directed antibodies persist for more than six months in a cohort with mild to moderate COVID-19

**DOI:** 10.1007/s15010-021-01598-6

**Published:** 2021-03-10

**Authors:** Vivian Glück, Sonja Grobecker, Leonid Tydykov, Bernd Salzberger, Thomas Glück, Tanja Weidlich, Manuela Bertok, Christine Gottwald, Jürgen J. Wenzel, André Gessner, Barbara Schmidt, David Peterhoff

**Affiliations:** 1 Institute of Clinical Microbiology and Hygiene, University Hospital Regensburg, Regensburg, Germany; 2 Department for Infection Control and Infectious Diseases, University Hospital Regensburg, Regensburg, Germany; 3 Kliniken Südostbayern, Traunstein, Germany; 4 Institute for Medical Microbiology and Hygiene, University of Regensburg, Regensburg, Germany

**Keywords:** SARS-CoV-2, COVID-19, Serological immune response, Antibody titer, ELISA, Severity of disease

## Abstract

**Objective:**

To follow serological immune responses of front-line healthcare workers after PCR-confirmed COVID-19 for a mean of 30 weeks, describe the time-course of SARS-CoV-2 spike protein-specific IgG, IgA and IgM levels and to identify associations of the immune response with symptoms, demographic parameters and severity of disease.

**Methods:**

Anti-SARS-CoV-2 S protein-specific IgG, IgA and IgM antibodies were measured at three time points during the 30-week follow-up. COVID-19-specific symptoms were assessed with standardized questionnaires.

**Results:**

95% of the participants mounted an IgG response with only modest decline after week 12. IgG-type antibodies were still detectable in almost 90% of the subjects at 30 weeks. IgA and IgM responses were less robust and antibody titers decreased more rapidly. At 30 weeks, only 25% still had detectable IgA-type and none had IgM-type antibodies. Higher age and higher disease severity were independently associated with higher IgG antibody levels, albeit with wide variations.

**Conclusion:**

Serological immune responses after COVID-19 show considerable inter-individual variability, but show an association with increasing age and higher severity of disease. IgG-type anti-SARS-CoV-2 antibodies remain positive in 90% of the individuals 30 weeks after onset of symptoms.

**Supplementary Information:**

The online version contains supplementary material available at 10.1007/s15010-021-01598-6.

## Introduction

The severe acute respiratory syndrome-related coronavirus 2 (SARS-CoV-2) was first isolated in January 2020 in the province of Wuhan, China, where clusters of severe atypical pneumonias with respiratory failure (COVID-19) had been noticed from December 2019 on [1, 2]. Rapidly spreading from China to other countries worldwide, this virus has since then caused an unprecedented pandemic with more than 95 million infected individuals and more than 2 million deaths so far, numbers still soaring [3].

Similar to other viral respiratory infections, patients infected by SARS-CoV-2 generally mount an immune response with virus-specific IgM, IgA and IgG antibodies, but anti-SARS-CoV-2 antibody titers appear to vary considerably between individuals [4–6]. Furthermore, it is so far unknown how long immunity against SARS-CoV-2 persists in patients who recovered from the infection. Previous investigations have shown that respiratory coronaviruses causing common colds usually elicit only weak immune responses that wane rapidly [7]. In contrast, immunity against the SARS-CoV-1 and MERS-coronaviruses that are more related to SARS-CoV-2, appear to be more sustained [8]. Investigations of the course of antibody responses against SARS-CoV-2 showed conflicting results so far. While some reports indicated rapidly waning antibody titers [9–11], others found a slower decline [6, 12, 13].

The purpose of this investigation was to describe the course of IgM, IgA and IgG antibody titers against SARS-CoV-2 over more than 6 months after infection in a well-characterized cohort of front-line healthcare workers with mild to moderate COVID-19 and to explore clinical parameters and infection-related symptoms that might be associated with the magnitude of the humoral response to SARS-CoV-2.

## Materials and methods

### Patient cohort

Individuals who participated in this study were employees of the Kliniken Südostbayern AG, a network of 6 hospitals that provides healthcare service for the southeastern region of Germany. All participants were diagnosed with COVID-19 by RT-qPCR (Roche Cobas 6800, Roche Diagnostics, Mannheim, Germany). According to health authority regulations, infected health care workers (HCWs) could only resume work with a negative nasopharyngeal swab at least 14 days after onset of symptoms or first positive test. HCWs with positive PCR tests at this time point were repeatedly tested every 3–5 days until negative. When the employees visited the Employee Health Department of the Kliniken Südostbayern AG for getting their SARS-CoV-2 PCR-test prior to resuming work, they were informed about the seroprevalence study and asked to participate. After written informed consent, 7 ml of blood was collected by venipuncture (S-Monovette, Sarstedt, Nümbrecht, Germany). Serum was obtained by centrifugation at 3000*g* for 10 min at room temperature. Samples were stored at − 20 °C until analysis by ELISA. Furthermore, the participants were asked to provide information about the nature, intensity and duration of symptoms (fever, nausea, diarrhea, loss of sense of smell or taste, fatigue, dyspnea, headache, cough, runny nose, sore throat and myalgia) related to their COVID-19 infection in a standardized questionnaire (see supplementary methods).

At approximately 12 and 24 weeks after enrollment in the study, participants were asked to donate a second and third serum sample. On the occasion of the donation of the third serum sample, they were asked to provide information about the duration of their symptoms in a second standardized questionnaire (see supplementary methods). Study participants did not receive any compensation or other benefit, but were informed individually about their antibody status. The study was approved by the ethical committee of the Faculty for Medicine, University of Regensburg, Regensburg, Germany (reference number 20-1896-101).

### Enzyme‑linked immunosorbent assay

Antibody concentrations were measured in an in-house enzyme‑linked immunosorbent assay (ELISA) as described previously [14]. Briefly, the ELISA uses the SARS-CoV-2 receptor-binding domain (RBD) as antigen and is able to detect IgM, IgA and IgG antibody responses with high specificity and sensitivity. The ELISA does not cross-react with seasonal coronavirus antibodies and correlates well with SARS-CoV-2 neutralization capacity of the serum. ELISA results were expressed as optical densities/background ratios (signal/cutoff; S/CO). ELISA readings ≤ 1.0 S/CO were considered negative. 1.0 < S/CO ≤ 3.0, 3.0 < S/CO ≤ 6.0, and S/CO > 6.0 were considered low, intermediate and high antibody titers, respectively. S/CO differences > 0.1 were defined as increase or decrease of the antibody level.

### Statistical analysis

Experimental data were analyzed using the SPSS software package (IBM SPSS Statistics Version 26, IBM, New York, USA). Associations between parameters were compared by *t*-test, Pearson’s product moment correlation or Spearman’s rank order correlation, where appropriate. The influence of time, disease severity (uncomplicated vs. moderate or severe disease) and age on anti-RBD IgG serum levels was examined in a generalized linear model with IgG over three time points as repeated measurements. Graphs were plotted using GraphPad Prism (GraphPad Prism for Windows version 9.0; GraphPad Software, San Diego/CA, USA). Locally weighted scatterplot smoothing was done using the LOWESS algorithm. A *p* value of *p* < 0.05 was considered significant.

## Results

### Demographics and characteristics of the study cohort

123 individuals participated in the study between April 8, 2020 and December 3, 2020. 75 (62%) were females (median age 40 years) and 46 (38%) were males (median age 37 years) (Fig. S1). 123, 83 and 123 participants donated serum samples during the first 8 weeks (median 3 weeks), between weeks 8 and 23 weeks (median 12 weeks), and between weeks 23 and 36 (median 30 weeks) after onset of symptoms. For temporal distribution of sampling dates, see Fig. S2. Information about severity of acute COVID-19-related symptoms was provided by 118 subjects and rated as mild, moderate or severe by 64 (54.2%), 44 (37.3%) and 10 (8.5%), respectively. 14/118 (11.9%) declared one or more comorbidities. No participant had to be admitted to hospital for inpatient treatment. 21 (17.8%), 23 (19.5%), 23 (19.5%) and 51 (43.2%) of the subjects showed acute symptoms for 0–3 days, 4–6 days, 7–9 days and > 10 days, respectively. Table 1 displays character and duration of the study participants' complaints (*n* = 119) indicated in the second questionnaire, obtained on the occasion of the third blood sampling 23–36 weeks after onset of symptoms.

**Table 1:** Character and duration of study participants’ symptoms (*N* = 119)

	Fever	Myalgia	Fatigue	Loss of sense of smell or taste	Diarrhea	Nausea	Cough	Dyspnea
Never	54 (45.4%)	33 (27.7%)	15 (12.6%)	38 (31.9%)	77 (64.7%)	94 (79.0%)	59 (49.6%)	47 (39.5%)
0–1 month	65 (54.6%)	67 (56.3%)	49 (41.2%)	35 (29.4%)	35 (29.4%)	22 (18.5%)	44 (37.0%)	31 (26.1%)
2–3 months	0 (0%)	10 (8.4%)	29 (24.4%)	21 (17.6%)	3 (2.5%)	3 (2.5%)	13 (10.9%)	11 (9.2%)
4–5 months	0 (0%)	2 (1.7%)	5 (4.2%)	2 (1.7%)	2 (1.7%)	0 (0%)	1 (0.8%)	11 (9.2%)
> 6 months	0 (0%)	7 (5.9%)	21 (17.6%)	23 (19.3%)	2 (1.7%)	0 (0%)	2 (1.7%)	19 (16.0%)

Total counts and percentage of subjects are given

### Anti-SARS-CoV-2 IgG responses

The first serum samples provided by the participants within 8 weeks after symptom onset showed no detectable anti-SARS-CoV-2 IgG antibodies (≤ 1.0 S/CO) in only 6 out of 123 (4.9%). Low (1.0 < S/CO ≤ 3.0), intermediate (3.0 < S/CO ≤ 6.0) and high (S/CO > 6.0) IgG antibody levels were found in 33/123 (26.8%), 49/123 (39.8%) and 35/123 (28.5%), respectively. Of the 6 participants with negative IgG titers within the first 8 weeks, three (of five evaluated) showed an increase to low (*n* = 2) and high (*n* = 1) antibody levels in the second samples; whereas, the other two remained seronegative. Two of these 6 individuals showed IgM and/or IgA titers, one a low IgM titer, the other intermediate IgA and IgM titers. In the third samples, 5 of the 6 subjects reverted to IgG negative, and one still showed an intermediate titer. Overall, the second and third blood samples had anti-SARS-CoV-2 IgG antibodies below the background threshold in 4/83 (4.8%) and 13/123 (10.6%), respectively.

High anti-SARS-CoV-2 IgG levels (> 6 S/CO) were found in 35/123 (28.5%) of participants in the first serum sample, but only in 23/83 (27.7%) and 15/123 (12.2%) of the second and third samples, respectively. Only one of the 35 individuals with high anti-SARS-CoV-2 IgG levels in the first sample reverted to seronegative in the third sample.

**Fig. 1:**
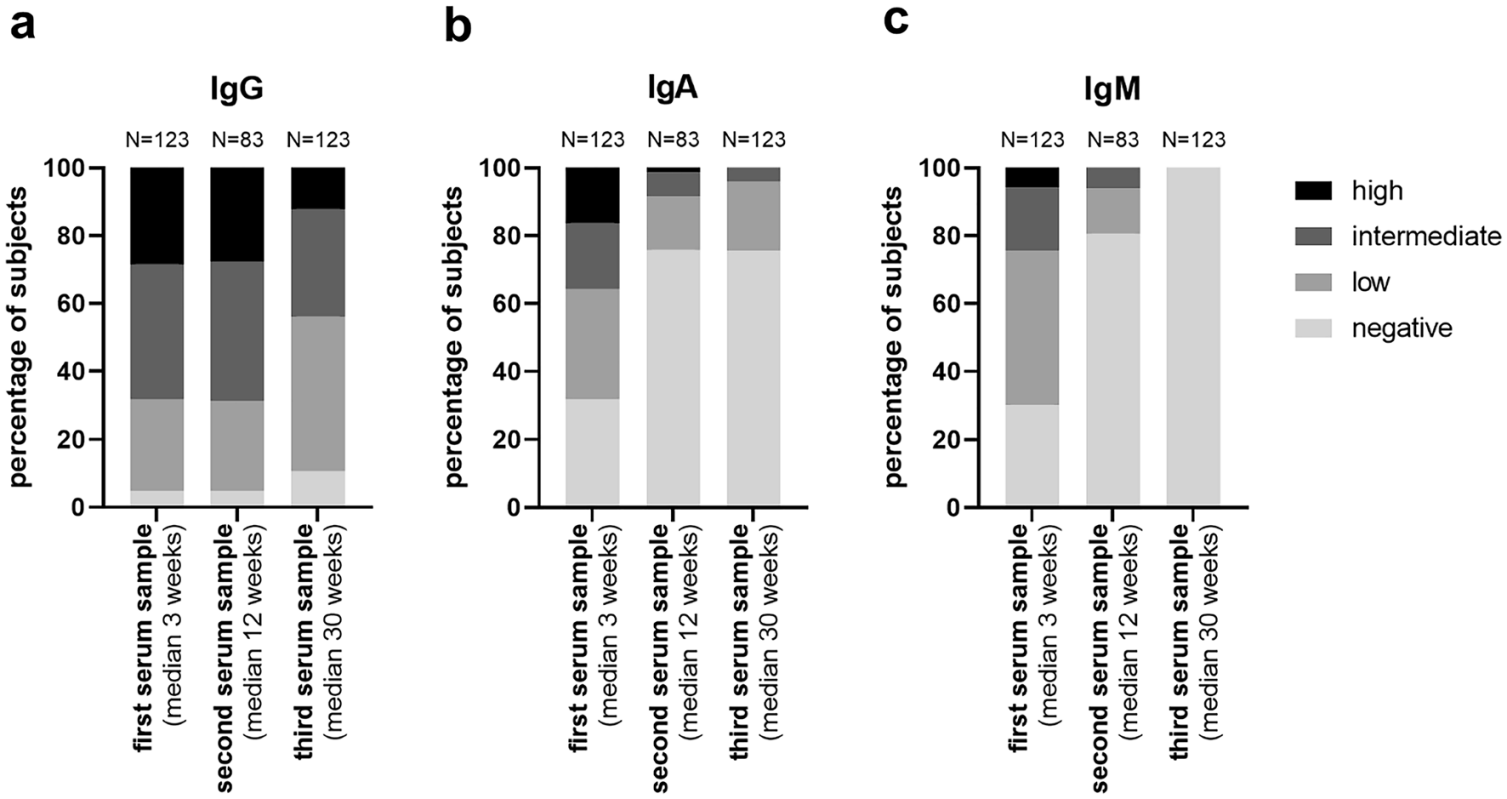
Time course of anti-SARS-CoV-2 S protein (RBD) directed antibody reactivities over 30 weeks. **a** IgG, **b** IgA and **c** IgM. Detailed information is provided in Table S1

**Fig. 2:**
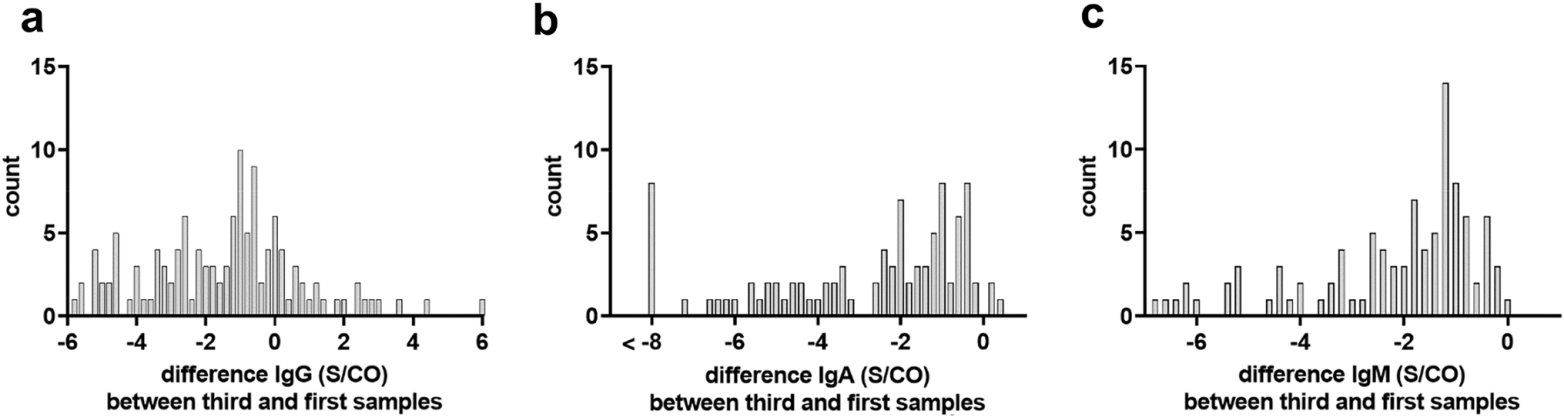
Relative differences of anti-SARS-CoV-2 IgG antibody titers between third and first samples. **a** IgG, **b** IgA and **c** IgM

30/83 (36.1%) showed increasing IgG levels from first to second samples, and 5/30 (16.7%) had a further increase from second to third samples; whereas, levels decreased in 25/30 (83.3%). In 22/123 (17.9%), anti-SARS-CoV-2 IgG levels in the third samples were higher than in the first. 44/83 (53.0%) had declining anti-SARS-CoV-2 IgG levels between first and second samples. Only 4/44 (9.1%) increased again thereafter from second to third samples (all with S/CO > 6.0), but the majority (39/44; 88.6%) declined further. In 9/83 (10.8%), anti-SARS-CoV-2 IgG levels did not change between first and second samples. The course of anti-SARS-CoV-2 IgG levels over time is displayed in Fig. 1a and listed in Tab. S1. The decay of anti-SARS-CoV-2 IgG antibody titers in the third relative to the first samples is displayed in Fig. 2a.

### Anti-SARS-CoV-2 IgA responses

Anti-SARS-CoV-2 IgA responses were generally weaker and shorter lived than IgG responses. The first serum samples showed negative, low, intermediate and high anti-SARS-CoV-2 IgA levels in 39/123 (31.7%), 40/123 (32.5%), 24/123 (19.5%), and 20/123 (16.3%), respectively. 53/83 (63.9%) individuals had declining IgA Titers between the first and second samples. 9/83 (10.8%) increased again slightly to low or intermediate IgA antibody levels from second to third samples, whereas 10/83 (12.0%) declined further. In the second samples, anti-SARS-CoV-2 IgA titers were negative, low, intermediate and high in 63/83 (75.9%), 13/83 (15.7%), 6/83 (7.2%) and 1/83 (1.2%), respectively. Among the third sample, 23–36 weeks after symptom onset, high IgA titers were not detected in any participant. Negative, low and intermediate anti-SARS-CoV-2 IgA levels were found in 93/123 (75.6%), 25/123 (20.3%) and 5/123 (4.1%), respectively. Those who were tested seronegative for anti-SARS-CoV-2 IgA in the first sample remained negative in the second and third samples. The distribution of anti-SARS-CoV-2 IgA levels over time is displayed in Fig. 1b, and the relative decay of levels from third relative to first sample in Fig. 2b.

### Anti-SARS-CoV-2 IgM responses

Absent, low, intermediate, and high anti-SARS-CoV-2 IgM titers were detected in 37/123 (30.1%), 56/123 (45.5%), 23 /123 (18.7%), and 7/123 (5.7%) of the first serum samples, respectively. Except for two individuals with mildly increasing anti-SARS-CoV-2 IgM titers from first to second samples, all others showed declines so that in the second samples negative, low and intermediate IgM titers were found in 67/83 (80.7%), 11/83 (13.3%), and 5/83 (6.0%), respectively. All individuals reverted negative for anti-SARS-CoV-2 IgM in the third samples. The distribution of anti-SARS-CoV-2 IgM levels over time is displayed in Fig. 1c, and relative decay of levels from third relative to first sample in Fig. 2c.

### Relationship between demographic parameters, severity of disease, and antibody responses

**Fig. 3:**
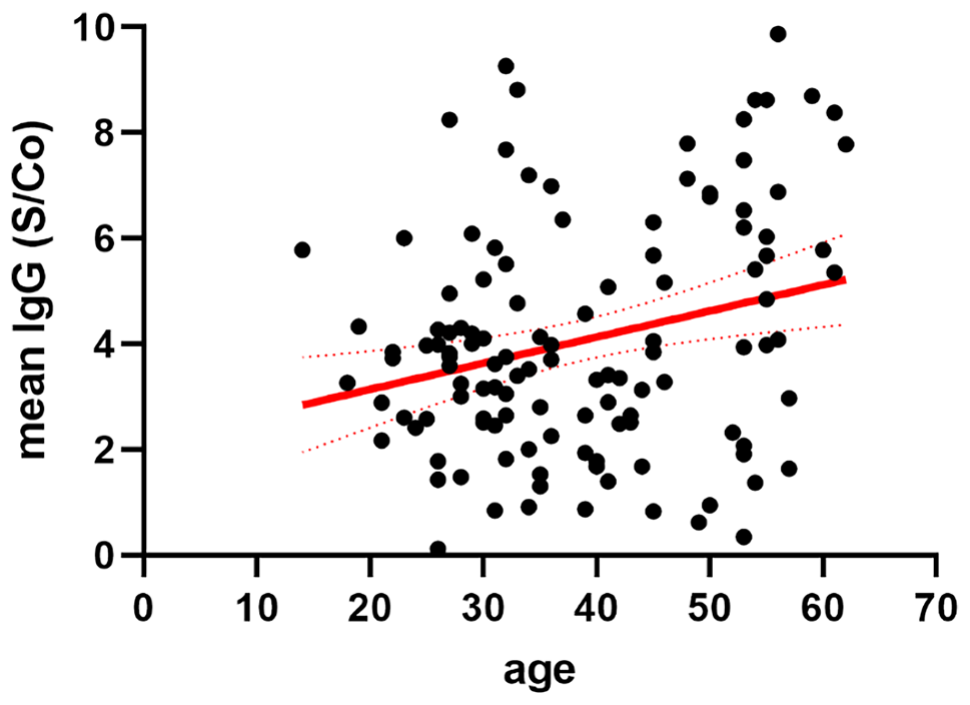
Correlation of age and mean anti-SARS-CoV-2 S protein-directed IgG antibody levels (*r* = 0.260; *p* = 0.004; regression line [solid] and 95% confidence interval [dotted] are given in red)

**Fig. 4:**
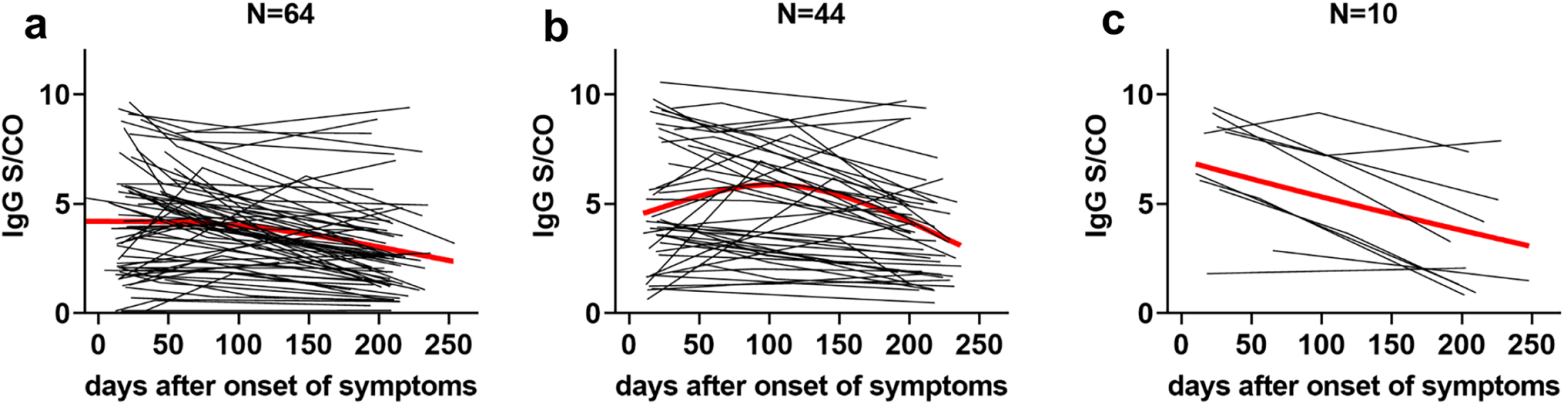
Course of anti-SARS-CoV-2 S protein-directed IgG antibody titers over time according to severity of symptoms. **a** mild, **b** moderate and **c** severe symptoms. General trend line is shown in red and was calculated using the LOWESS algorithm

IgG, IgA and IgM levels were highly significantly positively correlated with each other at the earlier time points of serum sampling (Fig. S3). Similarly, severity of disease, as indicated by the study subjects, was significantly positively correlated with duration of illness (*r* = 0.587; *p* < 0.001), comorbidities (*r* = 0.303; *p* = 0.001) and the individual symptoms (fever: *r* = 0.288; *p* = 0.002, myalgia: *r* = 0.319; *p* = 0.001, fatigue: *r* = 0.279; *p* = 0.003, diarrhea: *r* = 0.310; *p* = 0.001, nausea: *r* = 0.266; *p* = 0.004, cough: *r* = 0.357; *p* < 0.001 dyspnea: *r* = 0.346; *p* < 0.001). On univariate analysis, age was significantly, but mildly correlated with antibody levels of all classes at the first serum sampling (Fig. S3), severity of disease (*r* = 0.184; *p* = 0.046), and presence of any comorbidity (*r* = 0.270; *p* = 0.003). Figure 3 displays the correlation between age and means of the anti-SARS-CoV-2 IgG levels measured at first and third, or first, second and third samples, respectively (*r* = 0.260; *p* = 0.004). Severity of disease was also significantly, but mildly correlated with mean IgG levels (*r* = 0.194; *p* = 0.035). Figure 4 shows the time-course of anti-SARS-CoV-2 IgG levels in individuals with mild, moderate or severe disease. In a generalized linear model with IgG over three time points as repeated measures, IgG levels decrease significantly over time (*p* = 0.021). Furthermore, the model showed a significant association of IgG levels with age (*p* = 0.042) and a significant trend for higher IgG levels in individuals with more severe disease (*p* = 0.04 for mild vs. moderate disease severity, see Table 2). No significant associations were found between gender, duration of SARS-CoV-2-PCR positivity in nasopharyngeal swabs, or type or duration of symptoms and anti-SARS-CoV-2 antibody titers.

**Table 2:**
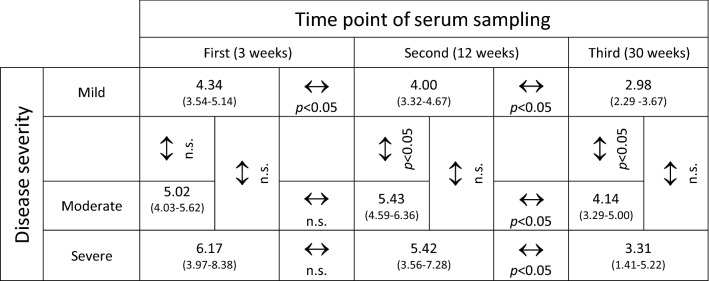
Results of a generalized linear model with IgG levels (mean and 95% confidence intervals) as dependent variable over three time points as repeated measurements

*n.s.* not significant

## Discussion

Several epidemiological investigations have shown that frontline healthcare workers are at significantly increased risk for acquiring COVID-19 infection [15, 16]. However, only few investigations focused so far on the course of the immune responses to SARS-CoV-2 in this population of usually healthy, mostly younger individuals during and after recovery from PCR-confirmed SARS-CoV-2 infection. In that respect, the cohort evaluated here differs from other published cohorts of patients with COVID-19 [17]. Determining the detailed natural course of anti-SARS-CoV-2 antibody titers in these convalescents with mostly relatively mild COVID-19 is of major relevance for estimating the long-term duration of protective immunity against SARS-CoV-2 in this constantly exposed, highly system relevant occupational group, and also for future comparisons with vaccination-induced immune responses in populations with few comorbidities.

Severity of disease in our cohort was generally mild according to standard criteria [18] (no participant had to be admitted to hospital or required oxygen supplementation), thus being in line with reports of approximately 90% of infections showing uncomplicated clinical progression [19]. Nevertheless, participants reported considerable morbidity and long-term sequelae. More than one out of five suffered from fatigue, dyspnea, and/or loss of sense of taste or smell for longer than 4 months after onset of symptoms. Similar frequencies of long-term sequelae have been reported previously [20].

The majority of individuals in this study mounted a serologic immune response, with anti-SARS-CoV-2 IgG antibodies detectable in more than 95% in the first serum samples obtained a median of 3 weeks after onset of symptoms. This seroconversion rate fits well with the 95% vaccine effectiveness rate observed in the recently published trial of the Comirnaty (BNT162b2, BioNTech) COVID-19 mRNA vaccine [21]. The IgG response measured in this study remained stable over the first 12 weeks and even some of the initial non-responders seroconverted late in weeks 8–23. After a median of 30 weeks, still almost 90% of the study participants had detectable anti-SARS-CoV-2 IgG titers with a modest decline in titers compared to weeks 3 and 12. Although some reports indicate that the immune responses to SARS-CoV-2 may wane rapidly [9, 11] and a considerable proportion of patients revert to seronegative [22], our results are in line with an increasing line of evidence showing that anti-SARS-CoV-2 IgG antibodies and virus neutralizing titers remain relatively stable or show only slow decay for at least 6 months [6, 12, 13, 23, 24]. These discrepancies may be explained in part by the use of different antigens or technologies in the various ELISAs. Self et al. [22], for example, used an ELISA that detects all immunoglobulin subclasses, while this study and others analyze IgG, IgA, and IgM responses separately. The S-protein’s RBD used here as ELISA antigen represents an important SARS-CoV-2 antigen and ELISA signals correlate well with virus neutralizing titers [14, 25]. Furthermore, robust T-memory cell responses over time were recently demonstrated in the majority of patients [12, 26]. Together with the persistence of antibody responses shown by us and others, this supports the concept of a longer-lived immunity against SARS-CoV-2.

The anti-SARS-CoV-2 IgA and IgM responses were less vigorous than the IgG responses in this study and showed a more rapid decline already after 12 weeks, which was even more pronounced after 30 weeks, when only 25% of the study participants still had IgA antibodies detectable and IgM antibodies had disappeared completely. The rapid loss of IgA-type antibodies also observed in other studies [6, 23] has raised concerns about loss of IgA-mediated mucosal immunity to SARS-CoV-2, thereby enabling localized SARS-CoV-2 infection with production of infectious viral particles, while the still present IgG-mediated systemic immunity impedes systemic symptoms so that such individuals might effectively transmit virus while being asymptomatic. This hypothesis, however, is yet to be proven but the observed rapid IgA decline could be in line with this notion.

Similar to previous reports, the magnitude of the anti-SARS-CoV-2 immune response in this study showed considerable inter-individual variability [27]. In this cohort with well-documented symptoms, we were unable to establish a relationship between character and duration of symptoms and antibody levels except for a moderate but significant association of age with levels of all antibody classes and overall severity of symptoms with higher anti-SARS-CoV-2 IgG levels. In contrast to the study by Choe et al. [24], we did not see a more vigorous antibody response among female participants.

Not all studies were able to demonstrate an association of disease severity with antibody titers [8, 28]. However, our observations are in line with an investigation of anti-SARS-CoV-2 neutralizing antibody levels and serological responses in two cohorts with disease characteristics similar to this study [24, 29] and in a study with generally sicker individuals [30]. To that extend, and considering that the ELISA used here correlates well with virus neutralization [14], our results rather support the hypothesis that a more vigorous immune response may, at the same time, cause more severe symptoms and generate higher (and possibly more durable) antibody titers. Interestingly, for so far unknown reasons also higher age appears to correlate with higher antibody levels. Since age and disease severity has been shown to correlate [31], higher titers be connected by this interrelation. However, a consensus how demographic parameters and severity of COVID-19 disease correspond with serological immune responses against SARS-CoV-2 has not been achieved so far.

In our study, for estimates of type and severity of symptoms, we relied on the participants' statements, which may be open for bias due to subjective perception, even though the majority of participants were medical professionals. Laboratory tests to complement the subjective grading were not available. However, the consistent correlations between symptoms and reported severity of disease indicate that the estimates of disease severity are reliable. Furthermore, we evaluated in this study only the serologic immune response to the SARS-CoV-2 RBD. Even though the RBD likely represents the most important structural target of SARS-CoV-2 for virus neutralizing antibodies and was chosen as primary antigen in current vaccines, this may be a limitation of this investigation since SARS-CoV-2 harbors many other immunogenic epitopes, some of which exhibit potentially beneficial cross-reactivity with other coronaviruses. Moreover, T-cell responses may be even more relevant than antibodies for the long-term protective immunity against SARS-CoV-2, and it is essentially unclear so far how serologic and T-cell responses to SARS-CoV-2 correspond [32].

In conclusion, this investigation shows that serological IgG responses against SARS-CoV-2 appear to persist for more than 6 months in the majority of individuals, and levels are mildly associated with age and severity of disease. Recently, sporadic cases of both symptomatic and asymptomatic reinfections have been described [33–36]. So far, we did not detect a symptomatic reinfection within our cohort. To what extend decreasing antibody titers after SARS-CoV-2 infection may lead to loss of protection and, thus, risk of reinfection is an important question to be addressed in future studies.

## Supplementary Information

Below is the link to the electronic supplementary material.

Supplementary file1 (DOCX 446 KB)
