# Supplementary material for: SARS-CoV-2-directed antibodies persist for more than six months in a cohort with mild to moderate COVID-19
Source: Infection. 2021 Mar 10;49(4):739–46. doi: 10.1007/s15010-021-01598-6 (PMC7944246; doi:10.1007/s15010-021-01598-6)
Supplement: Supplementary file 1 — Supplementary file1 (DOCX 446 KB) [file 15010_2021_1598_MOESM1_ESM.docx]

***Supplementary figures, tables and methods***

**SARS-CoV-2 directed antibodies persist for more than six months in a cohort with mild to moderate COVID-19**

Vivian Glück^1^, Sonja Grobecker^1^, Leonid Tydykov^1^, Bernd Salzberger^2^, Thomas Glück^3^, Tanja Weidlich^3^, Manuela Bertok^3^, Christine Gottwald^3^, Jürgen J Wenzel^1^, André Gessner^1,4^, Barbara Schmidt^1,4^, David Peterhoff^4^*

^1^Institute of Clinical Microbiology and Hygiene, University Hospital Regensburg, Regensburg, Germany

^2^Department for Infection Control and Infectious Diseases, University Hospital Regensburg, Regensburg, Germany

^3^Kliniken Südostbayern, Traunstein/Trostberg, Germany

^4^Institute for Medical Microbiology and Hygiene, University of Regensburg, Regensburg, Germany

*corresponding author: david.peterhoff@ur.de

**Supplementary Methods**

Questionnaire 1, distributed to participants on the occasion of study entry and donation of first blood sample, addressing acute symptoms.


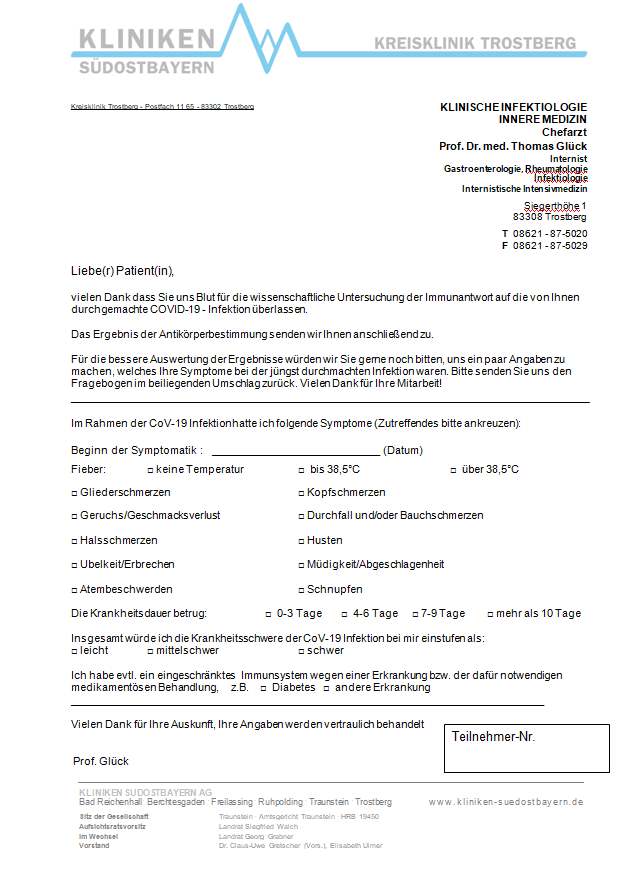


Questionnaire 2, distributed to participants on the occasion of donation of third blood sample, addressing long term symptoms.


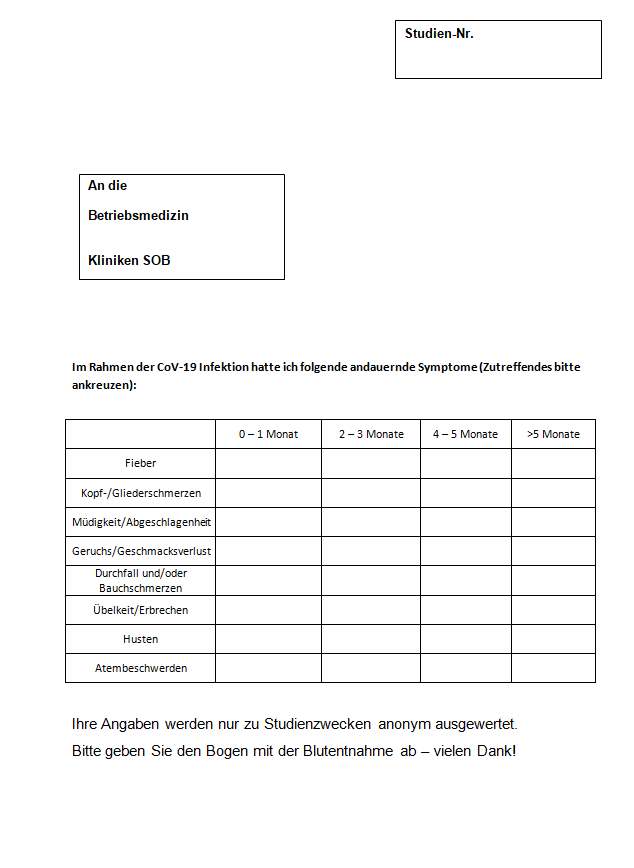


**Supplementary Tables**

**Tab. S1** Anti-SARS-CoV-2 antibody levels over 30 weeks. Fraction of total counts and percentage of subjects are given

|  | **time point** | **negative** | **low** | **intermediate** | **high** |
| --- | --- | --- | --- | --- | --- |
| **IgG** | 1-8 weeks | 6/123  (4.9%) | 33/123  (26.8%) | 49/123  (39.8%) | 35/123  (28.5%) |
|  | 8-23 weeks | 4/83  (4.8%) | 22/83  (26.5%) | 34/83  (41.0%) | 23/83  (27.7%) |
|  | 23-36 weeks | 13/123  (10.6%) | 56/123  (45.5%) | 39/123  (31.7%) | 15/123  (12.2%) |
| **IgA** | 1-8 weeks | 39/123  (31.7%) | 40/123  (32.5%) | 24/123  (19.5%) | 20/123  (16.3%) |
|  | 8-23 weeks | 63/83  (75.9%) | 13/83  (15.7%) | 6/83  (7.2%) | 1/83  (1.2%) |
|  | 23-36 weeks | 93/123  (75.6%) | 25/123  (20.3%) | 5/123  (4.1%) | 0  (0%) |
| **IgM** | 1-8 weeks | 37/123  (30.1%) | 56/123  (45.5%) | 23 /123  (18.7%) | 7/123  (5.7%) |
|  | 8-23 weeks | 67/83  (80.7%) | 11/83  (13.3%) | 5/83  (6.0%) | 0  (0%) |
|  | 23-36 weeks | 123/123  (100.0%) | 0  (0%) | 0  (0%) | 0  (0%) |

**Supplementary Figures**

**
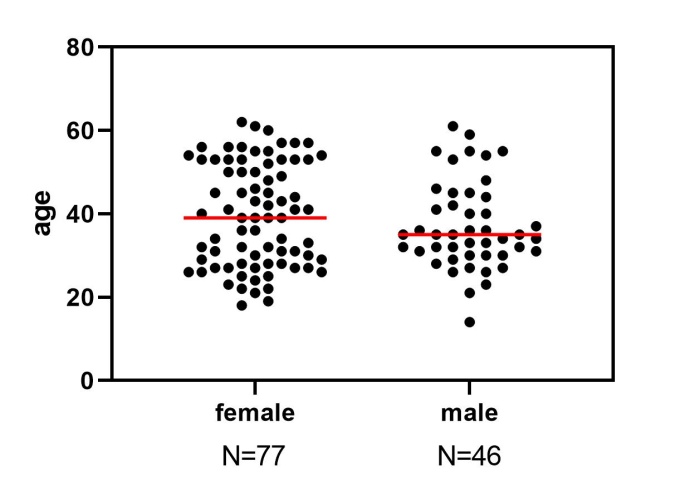
**

**Fig. S1** Study participants' sex and age distribution (median given in red)


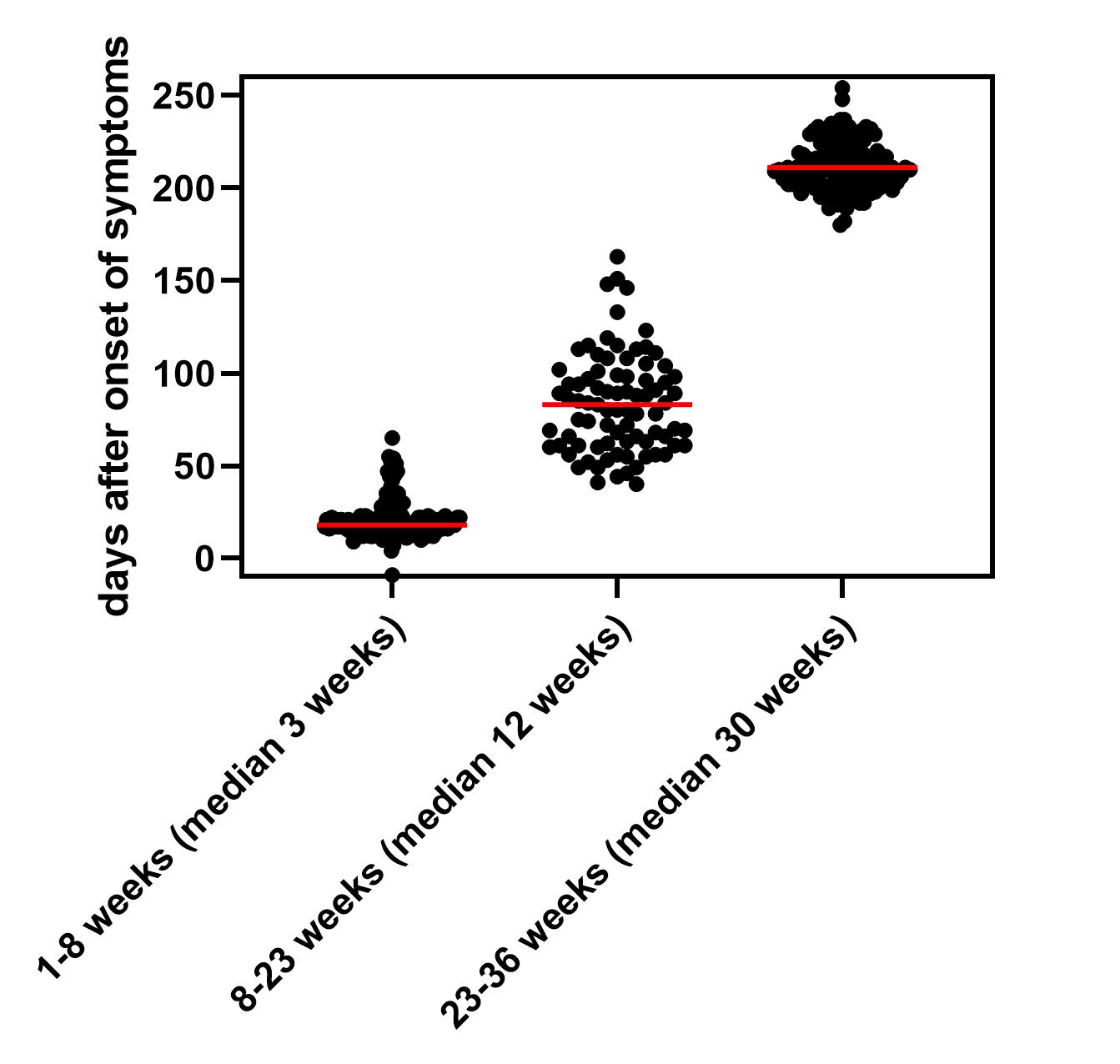


**Fig. S2** Distribution of sample collection time points relative to onset of COVID-19-symptoms (median given in red)


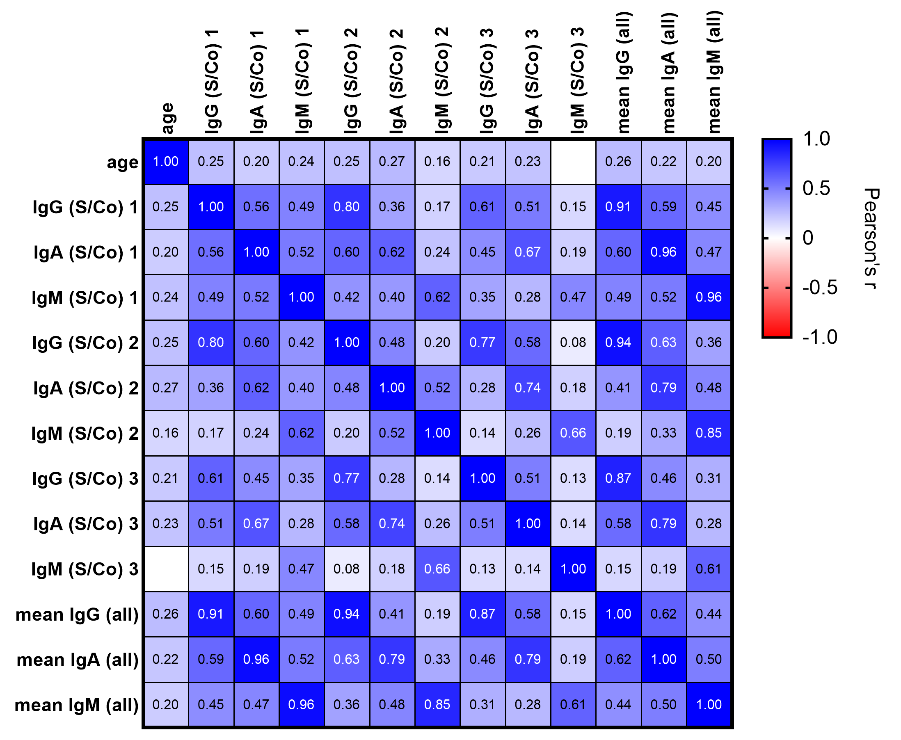


**Fig. S3** Correlation matrix of Pearson’s r values for the participants age and RBD ELISA reactivity (S/Co) for the three sampling time points (1, 2, 3) and the mean reactivity
